# Rituximab versus azathioprine as therapy for maintenance of remission for anti-neutrophil cytoplasm antibody-associated vasculitis (RITAZAREM): study protocol for a randomized controlled trial

**DOI:** 10.1186/s13063-017-1857-z

**Published:** 2017-03-07

**Authors:** Seerapani Gopaluni, Rona M. Smith, Michelle Lewin, Carol A. McAlear, Kim Mynard, Rachel B. Jones, Ulrich Specks, Peter A. Merkel, David R. W. Jayne, on behalf of the RITAZAREM Investigators

**Affiliations:** 1 University of Cambridge Hospitals NHS Foundation Trust, Cambridge, UK; 2 Division of Rheumatology and Department of Epidemiology and Biostatistics, University of Pennsylvania, Philadelphia, PA USA; 3 Department of Pulmonary and Critical Care Medicine, Mayo Clinic, Rochester, MN USA; 4 Addenbrooke’s Hospital, Box 118, Hills Road, Cambridge, CB2 0QQ UK

**Keywords:** ANCA-associated vasculitis, RITAZAREM, Rituximab, Azathioprine, Randomized clinical trial

## Abstract

**Background:**

Rituximab is effective as therapy for induction of remission in anti-neutrophil cytoplasmic antibody (ANCA)-associated vasculitis (AAV). However, the effect of rituximab is not sustained, and subsequent relapse rates are high, especially in patients with a history of relapse. There is a need to identify whether maintenance therapy with rituximab is superior to the current standard of azathioprine or methotrexate for prevention of relapse following induction with rituximab.

**Methods/design:**

RITAZAREM is an international, multicenter, open-label, randomized controlled trial designed to demonstrate the superiority of repeated doses of intravenous rituximab compared to daily orally administered azathioprine as a relapse prevention strategy in patients with AAV with relapsing disease who undergo induction of remission with rituximab. Patients with AAV will be recruited at the time of relapse and will receive rituximab and glucocorticoid induction therapy. If the disease is controlled by 4 months, patients will be randomized in a 1:1 ratio to receive rituximab (1000 mg every 4 months for five doses) or azathioprine (2 mg/kg/day) as maintenance therapy. Patients will be followed for a minimum of 36 months. The primary outcome is the time to disease relapse. It is estimated that 190 patients will need to be recruited to ensure that at least 160 are randomized.

**Discussion:**

The RITAZAREM trial will provide the largest trial dataset for the use of rituximab as remission-induction therapy for patients with AAV comparing two remission-maintenance strategies following induction with rituximab, and explore whether prolonged B-cell depletion leads to sustained treatment-free remissions after discontinuation of immunosuppressive therapy.

**Trial registration:**

ClinicalTrials.gov identifier: NCT01697267. Registered on 31 August 2012.

**Electronic supplementary material:**

The online version of this article (doi:10.1186/s13063-017-1857-z) contains supplementary material, which is available to authorized users.

## Background

Granulomatosis with polyangiitis (granulomatous with polyangiitis (GPA), Wegener’s granulomatosis) and microscopic polyangiitis (MPA) are two major subgroups of anti-neutrophil cytoplasmic antibody (ANCA)-associated vasculitis (AAV). These conditions are characterized by leukocyte infiltration of blood vessel walls, fibrinoid necrosis, and vascular damage, and are usually associated with the presence of circulating ANCA. In Europe, AAV has an annual incidence of 19 per million and a prevalence of 100–250/million [1].

Prior to the availability of effective treatment, AAV had a mortality of 93% within 2 years, primarily due to renal and respiratory failure [2]. The introduction of glucocorticoids (GC) and cyclophosphamide, now considered conventional immunosuppression for this disease, has markedly improved survival, inducing remission in approximately 80% of patients at 1 year. However, relapsing disease is common with over 50% of patients experiencing a relapse within 5 years [3–5].

Relapses of AAV lead to accrual of damage, both as a result of disease activity and increased exposure to immunosuppressive medications with their associated toxicities, especially GC, and at least 25% suffer severe early or late toxicity from these agents [6]. Strategies that prevent relapse will reduce consequent morbidity by reducing organ damage and cumulative drug toxicity. There is a need for safer therapies that lead to sustained, and ideally, drug-free remission in patients with relapsing disease in AAV.

B-lymphocytes have been implicated in the pathogenesis of AAV. Rituximab is a murine/human chimeric monoclonal antibody directed against the CD20 antigen found on the surface of B-lymphocytes, and results in B-cell depletion. Rituximab has been shown to be noninferior to cyclophosphamide as an induction agent, and it is now licensed for remission induction therapy in AAV [7, 8].

However, only 39% of the patients maintained relapse-free remission at 18 months following a single course of rituximab in the RAVE trial, indicating that induction with rituximab without maintenance therapy is unlikely to prevent future relapses in the majority of patients [9]. Retrospective analysis of fixed-interval, repeat-dose therapy with rituximab indicated efficacy of maintenance therapy with rituximab in this subgroup, but a persisting risk of relapse in longer-term follow-up after rituximab discontinuation [10–12].

### Rationale for the RITAZAREM trial

The MAINRITSAN trial was designed to compare four cycles of rituximab (500 mg × 2 at 6 months then 500 mg every 6 months × 3) with maintenance therapy with azathioprine following induction with cyclophosphamide in patients with AAV, largely with patients with newly diagnosed disease [13]. Fixed-interval rituximab was shown to be more effective in maintaining remission at 28 months. The MAINRITSAN trial has provided valuable information on the use of rituximab as a maintenance agent in AAV, although several important questions remain unanswered. Firstly, the MAINRITSAN study was underpowered to assess the effect of repeat-dose rituximab in the subgroup with the most need of a remission-maintenance strategy, namely patients with relapsing disease (only 23 of 115 patients had relapsing disease). Secondly, in many countries rituximab has now been granted a license for remission-induction in AAV, and is increasingly being used in place of cyclophosphamide. The optimal remission maintenance strategy following rituximab induction therapy is unknown. Thirdly, relapses do occur among patients treated with repeatedly-dosed rituximab, mostly in intervals of 6 more months [11, 12, 14]. Therefore, reducing the interval of repeated doses of rituximab to 4 months has the potential to further reduce relapse rates, and aims to maintain 100% B-cell depletion for the entire 24-month treatment phase. Finally, the total dose of rituximab for maintenance therapy in RITAZAREM is double that used in MAINRITSAN (5000 mg versus 2500 mg) which will permit comparison of the safety and longer-term efficacy of the two dosing regimens.

The RITAZAREM trial will address the question of the optimal treatment regimen for maintenance of remission following induction of remission with rituximab in AAV, and the possibility that prolonged B-cell depletion using high doses of rituximab, will lead, upon discontinuation of therapy, to sustained treatment-free remissions.

## Methods/design

RITAZAREM is an international, multicenter, open-label, randomized controlled trial designed to demonstrate the superiority of rituximab over azathioprine in the prevention of relapses in patients with AAV with relapsing disease (see Additional file 1 for the SPIRIT 2013 Checklist). This trial will also assess the risk of relapse after maintenance therapy is withdrawn at 24 months, the long-term safety of rituximab administration, and the optimal maintenance therapy in AAV following induction with rituximab.

The RITAZAREM trial has three phases:

1. An induction phase (months 0 to 4) in which eligible patients will be enrolled at the time of disease relapse and all will receive rituximab (4 weekly doses of 375 mg/m^2^) and GC
2. A maintenance phase (months 4 to 24): 4 months after enrollment, participants who achieve remission (defined as a Birmingham Vasculitis Activity Score for Wegener’s granulomatosis (BVAS/WG) ≤1 [15] and prednisone/prednisolone dose ≤10 mg/day) will be randomized in 1:1 ratio to receive 1000 mg rituximab at 4-monthly fixed intervals or daily azathioprine maintenance therapy (2 mg/kg/day)
3. A treatment-free follow-up period (minimum of 12, maximum of 24 months)

### Randomization and allocation

Enrollment and randomized allocation to treatment arm will be performed by an Internet-based website (TENELEA) provided by the Cambridge Clinical Trials Unit.

### Blinding

This is an open-label study. Several previous vasculitis studies that have had major influences on practice and have stood up to re-examination have been unblinded, and similar endpoints have been used, with processes developed in data management to check and manage potential bias. Blinding would have necessitated the administration of sham infusions and placebo tablet medications to all participants, which would have increased the complexity and cost of the trial and exposed participants to unnecessary interventions. A pragmatic approach of having the primary endpoint, relapse, independently assessed by expert adjudicators blinded to treatment allocation was decided.

### Participants

To be eligible, participants must be aged 15 years and above and should have a diagnosis of AAV according to Chapel Hill Consensus Conference definitions [16], along with a current or historical positive test for proteinase 3/myeloperoxidase anti-neutrophil cytoplasmic antibody (PR3/MPO ANCA) by enzyme-linked immunosorbent assay (ELISA). All patients must have disease relapse defined by one major or three minor disease activity items on the BVAS/WG and they must have previously achieved remission following at least 3 months of induction therapy, with a combination of GC and an immunosuppressive agent (cyclophosphamide, rituximab, methotrexate, or mycophenolate mofetil (MMF)).

Key exclusion criteria include the receipt of any biological B-cell-depleting agents within the previous 6 months, alemtuzumab, or anti-thymocyte globulin (ATG) within the last 12 months, or intravenously administered immunoglobulin (IVIg), plasma exchange, or anti-TNF treatment within the last 3 months. Patients with other multisystem autoimmune disease such as eosinophilic granulomatous with polyangiitis (eGPA), systemic lupus erythematosus (SLE), anti-glomerular basement membrane (GBM) disease or cryoglobulinaemic vasculitis, or history of malignancy within the past 5 years are also excluded. Additional file 2 lists the full inclusion and exclusion criteria for the study.

Participants will be recruited from many centers participating in the European Vasculitis Society (EUVAS), the Vasculitis Clinical Research Consortium (VCRC), and from centers in Japan, Australia, and New Zealand.

### Interventions

See Additional file 3 for figure: RITAZAREM trial flow chart.

#### Rituximab arm

*Induction phase*: rituximab 375 mg/m^2^/week × 4 doses. This dose (as opposed to the 2-weekly 1000-mg induction dose, which has been shown to be equivalent to the four-dose schedule in retrospective series [17]), was selected because it is the licensed induction regimen for AAV.

*Maintenance phase*: 1000 mg of rituximab repeated every 4 months for a total of five courses. Measureable B-cells usually return 4 months after a dose of rituximab, thus an every-4-month dosing interval aims to maintain 100% B-cell depletion for the entire 24-month treatment phase. Additionally, in one series of every 6-month fixed dosing of rituximab 90% of relapses occurred in 4–6 months following a dose of rituximab; therefore, reducing the interval between doses of rituximab has the potential to further reduce relapse rates [14]. There is no clear evidence of dose-dependent toxicity for rituximab, and thus an every-4-month schedule was chosen to maximize efficacy.

#### Control arm

*Induction phase*: rituximab 375 mg/m^2^/week × 4 doses.

*Maintenance phase*: orally administered azathioprine with a target dose of 2 mg/kg/day (maximum 200 mg/day) for 24 months. After 24 months, the dose will be reduced by 50% and it will be completely withdrawn at month 27.

Patients who are intolerant to azathioprine will receive either methotrexate (administered orally or subcutaneously) at a maximum dose of 25 mg/week (if estimated glomerular filtration rate is >50 ml/min), or MMF at a maximum dose of 2 g/day (if estimated glomerular filtration rate (GFR) is ≤50 ml/min).

### Concomitant therapy

#### Glucocorticoids

*Induction*: since evidence for optimal GC induction dosing in relapsing AAV is lacking, it was decided, to facilitate recruitment, to allow investigators to choose from one of two GC regimens to take into consideration variability in disease severity and local GC prescribing practices (Table 1). Patients may receive a maximum cumulative dose of 3000 mg intravenously administered (IV) methylprednisolone, between 14 days prior to enrollment and 7 days after enrollment.

**Table 1:** Orally administered glucocorticoid (prednisone/prednisolone) dosing in the RITAZAREM trial

	Induction schedule A (1 mg/kg group)		Induction schedule B (0.5 mg/kg group)	
Week	<60 kg	≥60 kg	<60 kg	≥60 kg
0	50 mg/day	60 mg/day	25 mg/day	30 mg/day
2	35 mg/day	45 mg/day	20 mg/day	25 mg/day
4	25 mg/day	35 mg/day	17.5 mg/day	20 mg/day
6	20 mg/day	25 mg/day	15 mg/day	17.5 mg/day
8	15 mg/day	17.5 mg/day	12.5 mg/day	15 mg/day
10	12.5 mg/day		12.5 mg/day	
12	10 mg/day		10 mg/day	

*Maintenance*: an orally administered GC (prednisone/prednisolone or the equivalent) dose of 10 mg/day or less is a requirement for randomization at 4 months. The GC dose is reduced to 5 mg/day by month 6, and is continued at 5 mg/day until month 16. The GC dose is then reduced to 2.5 mg/day and completely withdrawn at month 20.

### Other treatments

#### Prophylactic therapies

Prophylaxis to prevent *Pneumocystis* (*carinii*) *jiroveci* infection and/or to prevent osteoporosis are strongly recommended but implementation is left to local practice.

#### Plasma exchange

Plasma exchange can be administered during the induction period following local practice at the discretion of the investigator. Rituximab will not be administered within the 48 h prior to receiving a plasma exchange treatment.

### Treatment of relapse

Time to relapse is the primary endpoint of the study. Patients experiencing their first minor relapse after randomization and before month 24 will remain on their randomized treatment and the dose of orally administered prednisone/prednisolone will be increased to 20 mg/day for 1 week decreasing in daily 2.5-mg increments each week until the dose is back to the dose before the relapse at which point the patient returns to the standard dosing schedule. The second minor relapse, or first major relapse, occurring before month 24, will result in the patient being withdrawn from protocolized treatment and the patient will be treated according to the investigator’s discretion. Any relapse occurring after the 24-month treatment phase will be treated according to local best medical practice.

### Assessments

Evaluations (including clinical, biochemical, and patient-reported outcomes) will be performed at months 0, 1.5, 3, 4, 8, 12, 16, 20, 24, 27, 30, 36, and every 6 months until the last patient has completed 36 months in the study (see Additional file 4 – SPIRIT figure: RITAZAREM schedule of events). The maximum duration in the study for any individual is 48 months. Assessments will also be performed at the time of relapse. Data will be collected on paper Case Report Forms, and entered into an electronic database hosted by the Cambridge Clinical Trials Unit.

### Outcomes

#### Primary outcome

1. Time to disease relapse (either minor or major relapse), reported at 24 months

#### Secondary outcomes

1. Proportion of patients who maintain remission at 24 and 48 months
2. Time to a major or second minor relapse
3. Cumulative accrual of damage as measured by the Combined Damage Assessment (CDA) score [18]
4. Health-related quality of life as measured using 36-item Short Form Health Survey (SF-36), the European Quality of Life-5 Dimensions (EQ5D), and Patient Reported Outcomes Measurement Information System (PROMIS) short forms for fatigue, pain, physical function, and patient global assessment [19]
5. Cumulative GC exposure
6. Severe adverse event rates and infection (treated with either intravenously or orally administered antibiotics) rates

### Power calculation

Enrollment will be ongoing until 160 patients are randomized at their 4-month visits. It is anticipated that 190 patients will be required in order to randomize 160 patients. A power of 90% will be achieved under the alternative hypothesis of a hazard ratio of 0.42 at the 5% significance level with 58 observed relapses. Randomizing 160 patients will achieve this over the course of the study assuming a dropout rate up to 5% at 2 years and a relapse-free rate of 75% and 50% at 4 years in the experimental and control arms, respectively. The hazard ratio of 0.42 is based on assuming a relapse-free rate of 75% at 4 years in the rituximab arm versus 50% at 4 years in the standard treatment arm.

### Analyses

A statistical analysis plan, separate to the study protocol, will be finalized prior to any analyses being performed. The primary intention-to-treat analysis will be based on a Cox proportional hazard model. There will be a closed testing procedure. Hazard ratios and 95% confidence intervals will be reported. First, the null hypothesis will be for a hazard ratio of 1 at all time points. If this is rejected at a 5% level then two further subhypotheses will be examined using time-varying covariates:

1. A hazard ratio of 1 up to 24 months post randomization (i.e. during treatment)
2. A hazard ratio of 1 after 24 months post randomization (i.e. post treatment)

### Funding, sponsor, and committee oversight

RITAZAREM is funded by grants from Arthritis Research UK and Roche/Genentech. The Vasculitis Clinical Research Consortium (VCRC) (U54 AR057319 and U01 AR5187404) is part of the Rare Diseases Clinical Research Network (RDCRN), an initiative of the Office of Rare Diseases Research (ORDR), National Center for Advancing Translational Science (NCATS). The VCRC is funded through collaboration between NCATS, and the National Institute of Arthritis and Musculoskeletal and Skin Diseases, and has received funding from the National Center for Research Resources (U54 RR019497). The primary sponsor is Cambridge University Hospitals NHS Foundation Trust and there are collaboration and data-sharing agreements with the University of Pennsylvania and the University of Miyazaki Hospital and Okayama University Graduate School of Medicine, Dentistry, and Pharmaceutical Sciences in Japan. A NIAMS Data and Safety Monitoring Board (DSMB) will provide oversight of this study. This is a standing DSMB that provides such oversight for all VCRC research studies, including randomized trials. The DSMB will meet at least every 6 months to discuss the study progress and more frequently, if needed. The Trial Steering Committee will provide overall supervision of the study on behalf of the principal funder, Arthritis Research UK. The Trial Steering Committee will monitor the progress of the study and maximize the chances of completing the study within the agreed time scale and budget. The Trial Management Committee is responsible for the design, conduct and overall management of the study. Cambridge Clinical Trials Unit has provided support in all phases of trial design, conduct, and management for this trial.

### Patient participation

The design of the study was presented at multiple local, national, and international meetings with patient attendees with positive review and input received from patients with AAV. The Patient Information Sheet was reviewed and commented upon by a patient panel prior to submission to the Ethics Committee.

## Discussion

It is becoming increasingly clear that patients with AAV with relapsing disease need different treatment strategies compared to patients with nonrelapsing disease. RITAZAREM is the largest clinical trial recruiting only the subgroup of patients with relapsing AAV.

The RITAZAREM trial will add to our knowledge on the use of rituximab both as an induction and a maintenance therapy in this particular patient population. This trial will also be the largest study to examine the outcome of induction with rituximab at 4 months in patients with relapsing AAV.

The MAINRITSAN study has shown that fixed-interval re-dosing of rituximab is effective in patients with AAV with new or relapsing disease but the RITAZAREM trial will also answer the following specific questions:

- Is fixed-interval rituximab re-dosing therapy superior to azathioprine in maintaining remission following rituximab induction in relapsing patients?
- Does a higher rituximab dose confer long-term benefit after discontinuation of therapy, without significant drug-related toxicity?

Data generated from the RITAZAREM study will compliment the already-available results of the MAINRITSAN trial [13] and published cohort studies [11, 12, 14]. RITAZAREM is an example of an academic-led clinical trial in a rare disease, facilitated by highly collaborative work of established regional vasculitis networks in Europe, North America, Japan, and Australia/New Zealand. It also demonstrates collaboration between academia and the pharmaceutical industry and continues the repurposing of a drug originally developed for lymphoma but subsequently licensed for the autoimmune disease rheumatoid arthritis.

### Trial status

Enrollment was completed on 21 November 2016, and thus randomization will be complete by the end of March 2017. The primary endpoint will be reported in 2019, and the final trial report in 2020.

## Additional files

Additional file 1: SPIRIT_Fillable-checklist-RITAZAREM; populated SPIRIT Checklist. (DOC 122 kb)
Additional file 2: Inclusion and exclusion criteria for RITAZAREM trial; full list of enrollment requirements. (DOC 34 kb)
Additional file 3: RITAZAREM trial flow chart; RITAZAREM study schema. (DOC 68 kb)
Additional file 4: SPIRIT figure: RITAZAREM schedule of events; populated SPIRIT figure. (DOC 75 kb)

## Abbreviations

AAV: ANCA-associated vasculitis
ANCA: Anti-neutrophil cytoplasmic antibody
BVAS: Birmingham Vasculitis Activity Score
CDA: Combined Damage Assessment
DSMB: Data Safety Monitoring Board
eGPA: Eosinophilic granulomatous with polyangiitis
ELISA: Enzyme-linked immunosorbent assay
EQ5D: European Quality of Life-5 Dimensions
EUVAS: European Vasculitis Society
GBM: Glomerular basement membrane
GC: Glucocorticoids
GFR: Glomerular filtration rate
GPA: Granulomatous with polyangiitis
IV: Intravenous
IVIg: Intravenous immunoglobulin
MMF: Mycophenolate mofetil
MPA: Microscopic polyangiitis
MPO: Myeloperoxidase
NIAMS: The National Institute for Arthritis and Musculoskeletal and Skin Diseases
PR3: Proteinase 3
PROMIS: Patient Reported Outcomes Measurement Information System
SF36: Short Form 36
SLE: Systemic lupus erythematosus
VCRC: Vasculitis Clinical Research Consortium
