# Supplementary material for: Rituximab versus azathioprine as therapy for maintenance of remission for anti-neutrophil cytoplasm antibody-associated vasculitis (RITAZAREM): study protocol for a randomized controlled trial
Source: Trials. 2017 Mar 7;18:112. doi: 10.1186/s13063-017-1857-z (PMC5341185; doi:10.1186/s13063-017-1857-z)
Supplement: Additional file 2: — Inclusion and exclusion criteria for RITAZAREM trial; full list of enrollment requirements. (DOC 34 kb) [file 13063_2017_1857_MOESM2_ESM.doc]

Additional file 2: Inclusion and exclusion criteria for RITAZAREM trial

**Inclusion Criteria**

To be included in the trial the patient must have:

1. Age ≥ 15 years
2. Provided written informed consent (15 years and above)
3. A diagnosis of AAV [granulomatosis with polyangiitis or microscopic polyangiitis], according to the definitions of the Chapel Hill Consensus Conference(16)
4. Current or historical positive test for PR3/MPO ANCA by ELISA
5. Disease relapse defined by one major or three minor disease activity items on the Birmingham Vasculitis Activity Score for Wegener’s (BVAS/WG), in patients that previously achieved remission following at least 3 months of induction therapy, with a combination of glucocorticoids and an immunosuppressive agent (cyclophosphamide or methotrexate or rituximab or mycophenolate mofetil)

**Exclusion Criteria**

1. Age < 15 years

2. Exclusions related to medication:

Previous therapy with:

- 1. Any biological B cell depleting agent (such as rituximab or belimumab) within the past 6 months
  2. Alemtuzumab or anti-thymocyte globulin (ATG) within the last 12 months
  3. Abatacept, adalimumab, etanercept, infliximab, intravenous immunoglobin or plasma exchange in past 3 months
  4. Any investigational agent within 28 days of screening, or 5 half lives of the investigational drug (whichever is longer)

3. Exclusions related to general health:

- 1. Significant or uncontrolled medical disease not related to AAV, which in the investigators opinion would preclude patient participation
  2. Presence of another multisystem autoimmune disease, including Churg Strauss syndrome, systemic lupus erythematosus, anti-GBM disease, or cryoglobulinaemic vasculitis,
  3. Any concomitant condition anticipated to likely require greater than 4 weeks per year of oral or systemic glucocorticoid use and which would preclude compliance with the glucocorticoid protocol (e.g. poorly controlled asthma, COPD, psoriasis, or inflammatory bowel disease).
  4. History of severe allergic or anaphylactic reactions to humanised or murine chimeric monoclonal antibodies
  5. Known infection with HIV (HIV testing will not be a requirement for trial entry); a past or current history of hepatitis B virus or hepatitis C virus infection.
  6. Ongoing or recent (last 12 months) evidence of active tuberculosis or known active infection (screening for tuberculosis is part of ‘standard of care’ in patients with established AAV) or evidence of untreated latent tuberculosis. Screening for tuberculosis is as per local practice.
  7. History of malignancy within the past five years or any evidence of persistent malignancy, except fully excised basal cell or squamous cell carcinomas of the skin, or cervical carcinoma in situ which has been treated or excised in a curative procedure.
  8. Pregnancy or inadequate contraception in women of childbearing potential
  9. Breast feeding or lactating
  10. Medical, psychiatric, cognitive or other conditions that, in the investigator's opinion, compromise the patient's ability to understand the patient information, to give informed consent, to comply with the trial protocol, or to complete the study.

4. Exclusion criteria related to laboratory parameters:

- 1. Bone marrow suppression as evidenced by a total white count < 4 x10^9^/l, haemoglobin < 7 gm/dl or platelet count < 100,000/μl

Aspartate aminotransferase or alanine aminotransferase or amylase > 2.5 times the upper limit of normal, unless attributed to vasculitis
