# Supplementary material for: Rituximab versus azathioprine as therapy for maintenance of remission for anti-neutrophil cytoplasm antibody-associated vasculitis (RITAZAREM): study protocol for a randomized controlled trial
Source: Trials. 2017 Mar 7;18:112. doi: 10.1186/s13063-017-1857-z (PMC5341185; doi:10.1186/s13063-017-1857-z)
Supplement: Additional file 3: — RITAZAREM trial flow chart; RITAZAREM study schema. (DOC 68 kb) [file 13063_2017_1857_MOESM3_ESM.doc]

**Additional file 3:** RITAZAREM Trial Flow Chart

4 month induction phase

**Screening and Enrolment**

Induction therapy with rituximab (4 x 375 mg/m^2^) and glucocorticoids (GC)

**Randomization**

At 4 months for patients demonstrating disease control (BVAS/WG ≤ 1 and GC dose ≤ 10mg per day)

20 month remission phase

**Rituximab Maintenance**

**Azathioprine Maintenance**

1000mg at 4, 8, 12, 16 & 20 months

Standardised GC taper

2mg/kg/day

Standardised GC taper

**Follow-Up Phase**

12-24 month follow-up phase

**Follow-Up Phase**

Azathioprine withdrawal month 27.

Follow-up between 36 months (min) and 48 months (max)

No therapy.

Follow-up between 36 months (min) and 48 months (max)
