# Supplementary material for: Rituximab versus azathioprine as therapy for maintenance of remission for anti-neutrophil cytoplasm antibody-associated vasculitis (RITAZAREM): study protocol for a randomized controlled trial
Source: Trials. 2017 Mar 7;18:112. doi: 10.1186/s13063-017-1857-z (PMC5341185; doi:10.1186/s13063-017-1857-z)
Supplement: Additional file 4: — SPIRIT figure: RITAZAREM schedule of events; populated SPIRIT figure. (DOC 75 kb) [file 13063_2017_1857_MOESM4_ESM.doc]

|  | **STUDY PERIOD** | | | | | | | | | | | | | | |
| --- | --- | --- | --- | --- | --- | --- | --- | --- | --- | --- | --- | --- | --- | --- | --- |
|  | **Enrolment** | | | | **Allocation** | **Post-allocation** | | | | | | | | **Close out** | |
|  | **Induction phase** | | | | | **Maintenance phase** | | | | | **Follow up phase** | | | | |
| **M=Month** | **Screen** | ****M0** | **M1.5** | **M3** | **M4** | **M8** | **M12** | **M16** | **M20** | **M24** | **M27** | **M30** | **M36** | **M42** | **M48** |
| **Enrolment** |  | | | | | | | | | | | | | |  |
| Eligibility Review | X |  |  |  |  |  |  |  |  |  |  |  |  |  |  |
| Informed Consent | X |  |  |  |  |  |  |  |  |  |  |  |  |  |  |
| Demographics | X |  |  |  |  |  |  |  |  |  |  |  |  |  |  |
| Weight | X |  |  |  | X |  | X |  |  | X |  |  | X |  |  |
| Height | X |  |  |  |  |  |  |  |  |  |  |  |  |  |  |
| Baseline medical history/co-morbidities | X |  |  |  |  |  |  |  |  |  |  |  |  |  |  |
| Randomisation |  |  |  |  | X |  |  |  |  |  |  |  |  |  |  |
| **Interventions** |  | | | | | | | | | | | | | |  |
| Rituximab induction (all patients)  **There are visits at weeks 1, 2, 3 and 4 for rituximab infusions |  | X |  |  |  |  |  |  |  |  |  |  |  |  |  |
| Rituximab maintenance |  |  |  |  | X | X | X | X | X |  |  |  |  |  |  |
| Azathioprine maintenance |  |  |  |  | X | X | X | X | X | X |  |  |  |  |  |
| **Assessments** |  | | | | | | | | | | | | | |  |
| Follow up |  |  | X | X | X | X | X | X | X | X | X | X | X | X | X |
| Concomitant meds | X | X | X | X | X | X | X | X | X | X | X | X | X | X | X |
| BVAS/WG (disease activity) |  | X | X | X | X | X | X | X | X | X | X | X | X | X | X |
| CDA (damage assessment) |  | X |  |  | X |  | X |  |  | X |  |  | X | X | X |
| SF-36, EQ5D (quality of life measures) |  | X |  |  | X |  | X |  |  | X |  |  | X | X | X |
| PROMIS questionnaires |  | X | X | X | X | X | X | X | X | X | X | X | X | X | X |
| Adverse event review |  | X | X | X | X | X | X | X | X | X | X | X | X | X |  |
| Clinical labs (FBC/CBC; ESR; chemistry, CRP) | X | X | X | X | X | X | X | X | X | X | X | X | X | X | X |
| ANCA | X | X | X | X | X | X | X | X | X | X | X | X | X | X | X |
| Urinalysis | X | X | X | X | X | X | X | X | X | X | X | X | X | X | X |
| Lymphocyte markers |  | X | X | X | X | X | X | X | X | X | X | X | X | X | X |
| Immunoglobulins |  | X | X | X | X | X | X | X | X | X | X | X | X | X | X |
| Research specimens (Serum; plasma) |  | X |  |  | X | X | X | X | X | X | X | X | X | X | X |
| DNA |  | X |  |  |  |  |  |  |  |  |  |  |  |  |  |
| RNA |  | X |  |  | X |  |  |  |  | X |  |  | X |  |  |

BVAS/WG - Birmingham Vasculitis Activity Score for Wegener’s Granulomatosis; CDA – combined damage assessment; SF-36 – Short form 36; EQ5D - European Quality of Life-5 Dimensions; PROMIS - Patient Reported Outcomes Measurement Information System; ANCA - Anti-neutrophil cytoplasmic antibody.
